# The Anatomy of the Brain

**Published:** 1900-09

**Authors:** 


THE ANATOMY OF THE BRAIN. A Text-book for Medical Students. By RICHARD H. WHITEHEAD, M. D., Professor of Anatomy in the University of North Carolina. Illustrated with Forty-one Engravings. Pages, v—96. The F. A. Davis Co., Publishers, 1914–16 Cherry Street, Philadelphia, Pa. [Price, extra vellum cloth, $1.00 net.]

The author does not claim that this little book shall take the place of the recognised standard works on the brain and nervous system, but it is his aim to furnish medical students with a clear, accurate and concise account of the anatomy of the brain, to be used as a guide in the study of this most important organ. It is not to be expected that in a work of ninety-six pages the subject of brain anatomy could be treated without leaving out many of the minor details, the points over which controversy still exists, and the minute anatomy or histology, and the author wisely draws attention to these omissions in the preface.

The author describes the intricate details of brain anatomy clearly and lucidly, and although briefly, yet sufficiently to give a good idea of the mechanism of the human brain.

On the whole, the book gives promise of becoming an excellent treatise with succeeding editions, and the hope is expressed that the author may become imbued with the prevalent idea of expansion and take up some of the minor and controversial points, and develop them into a treatise sufficiently advanced for the general practitioner and specialist. W. C. K.

